# 3D assessment of the nasolabial region in cleft models comparing an intraoral and a facial scanner to a validated baseline

**DOI:** 10.1038/s41598-023-39352-7

**Published:** 2023-07-27

**Authors:** Manuel Olmos, Ragai Matta, Mayte Buchbender, Fabian Jaeckel, Christopher-Philipp Nobis, Manuel Weber, Marco Kesting, Rainer Lutz

**Affiliations:** 1 Department of Oral and Cranio-Maxillofacial Surgery, Friedrich-Alexander-Universität Erlangen-Nürnberg, Glueckstrasse 11, 91054 Erlangen, Germany; 2 Department of Prosthodontics, Friedrich-Alexander-Universität Erlangen-Nürnberg, Erlangen, Germany

**Keywords:** Medical research, Paediatric research

## Abstract

We aimed to validate the metric accuracy of a 3-dimensional (3D) facial scanner (FS) and an intraoral scanner (IOS) in capturing the nasolabial region in ex vivo unilateral cleft lip and palate (UCLP) models. The nasolabial region of 10 UCLP models was scanned using a 3D FS as well as an IOS and a previously validated stationary 3D scanner as a reference. Intraoral scan was performed directly on the UCLP models. In order to apply the FS on the models, they were embedded in a 3D printed sample face. Both test groups were aligned to the reference by applying a section-based best-fit algorithm. Subsequent analysis of the metric deviation from the reference was performed with a 3D analysis tool. Mean distance and integrated distance served as main parameters for surface and volume comparison. Point comparison served as an additional parameter. Statistical analysis was carried out using t-test for unconnected samples. Considering mean distance and integrated distance as main parameters for 3D evaluation of the scanner’s accuracy, FS and IOS differ significantly in their metric precision in scanning the cleft model compared to the reference. The IOS proved to be significantly more accurate than the FS compared to the previously described stationary 3D scanner as reference and validated baseline. Further validation of the tested IOS and FS for 3D assessment of the nasolabial region is presented by adding the previously validated ATOS III Triple Scan blue light scanner as a reference. The IOS shows, compared to a validated baseline scan, significantly higher metric precision in experimental cleft model scanning. The collected data provides a basis for clinical application of the IOS for 3D assessment of the nasolabial region.

## Introduction

Cleft surgery usually results in severe functional and aesthetic changes at a young age, followed by a range of long-term needs in adulthood^1^. Appropriate methods are crucial for the long-term evaluation of postoperative outcomes and thus for the surgeon's ability to adapt his methods to specific patients and their anatomical variations. The introduction of accurate 3D documentation through modern assessment methods would make the long-term effects of various surgical techniques more tangible to young colleagues and thus more predictable at an earlier stage.

Until now, almost all evaluations are based on subjective assessments or measurements of 2-dimensional patient photographs^2–4^. However, literature indicates that 3D imaging appears to be most reliable^5,6^. High metric precision and sufficient mobility are two main parameters for the practicability of a 3D scanner in clinical-scientific applications. A metrically accurate method for 3D registration was previously presented for cast cleft models by comparing direct measurement to indirect 2D and 3D image measurements^6^. Since the accuracy of 3D surface photogrammetry was almost as good as that of direct anthropometry and significantly better than that of 2D photogrammetry, feasibility of the tested 3D photogrammetry methods and superiority over 2D methods for craniofacial anthropometric examinations was demonstrated. However, as the 3D scanner tested, as well as the previously validated scanner used as a reference in this study, is a stationary device, its practicability in a clinical setting must be considered limited.

Appropriate diagnostic methods for long-term assessment must not only be precise, but also as minimally invasive as possible, as radiation exposure plays a major role in adolescents^7,8^. Both IOS and FS work on the basis of optical recording methods and can therefore be classified as harmless in terms of radiation protection. This makes their use for regular follow-ups feasible even for very young patients. Practicability of new measurement techniques has top priority in clinical routine. Since conventional 2D measurement techniques in the form of patient images are routinely produced in most clinics^5^, innovative techniques must present comparable time efficiency and applicability. The aim of this study is to validate a mobile and therefore intraoperatively applicable scanner as a possible tool for the assessment of the nasolabial region in cleft patients through an ex vivo and therefore practical study design. In order to provide validated surface scans for accurate pre- and post-operative evaluation of cleft lip and palate surgery, a FS (Canfield, Vectra H2) and an IOS (3Shape, Trios 4) were compared with a previously validated stationary and therefore clinically inapplicable scanner (Co. GOM, ATOS Triple Scan) using a 3D evaluation and measurement tool (Co. GOM, GOM Inspect).

## Methods

### UCLP models and scanning process

**Figure 1:**
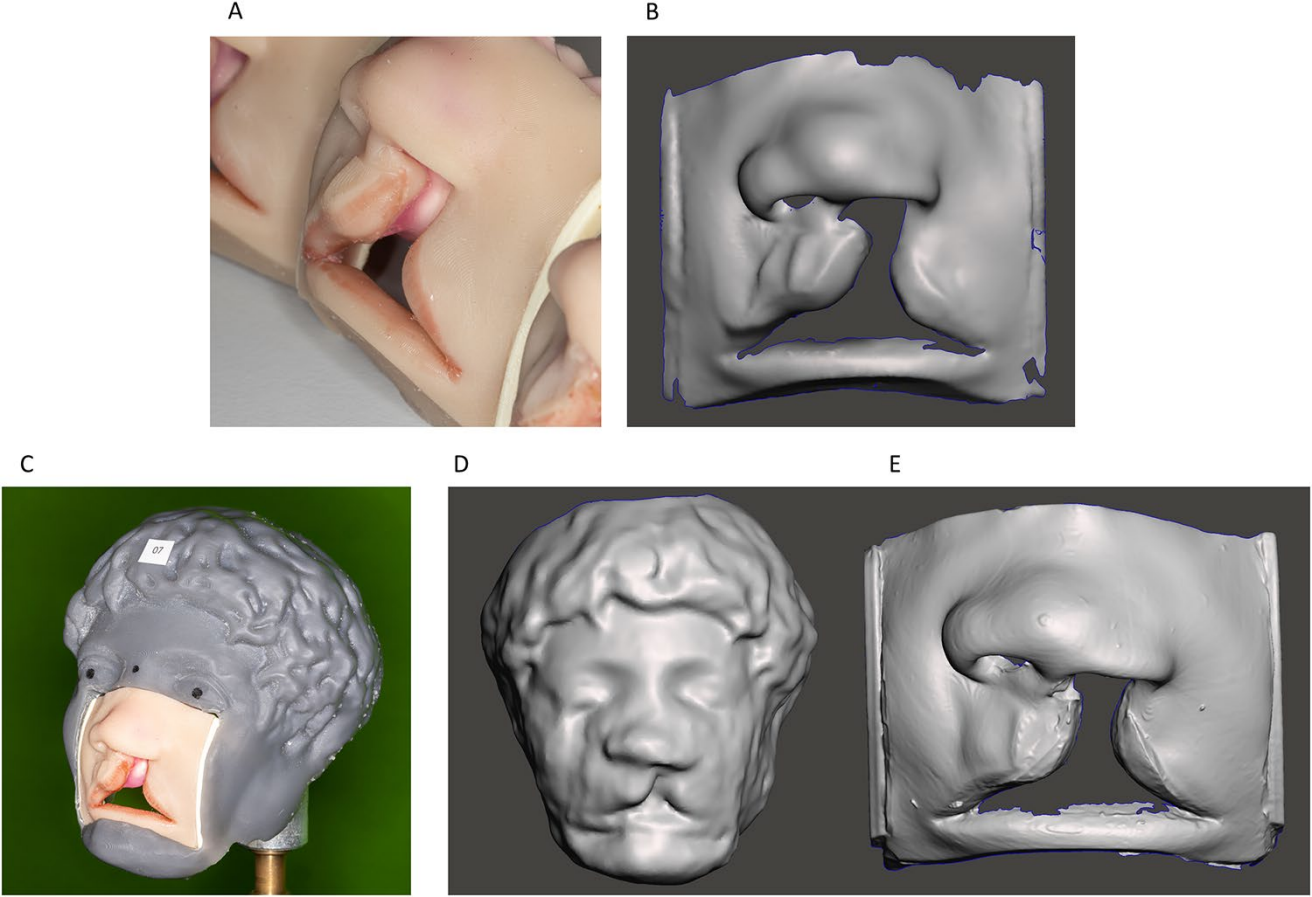
UCLP models, 3D printed sample face and the corresponding 3D scans. (**A**) UCLP models (Co. Smile Train, Cleft Lip Simulator). (**B**) Reference scan obtained by using a high precision previously validated non-contact optical blue-light scanner (Co. GOM, ATOS III Triple Scan). (**C**) UCLP model embedded in a 3D-printed sample face individually designed and printed to enable facial scan on the models. (**D**) Test scan obtained by using a facial scanner (Co. Canfield, Vectra H2) with a model embedded in the sample face. (**E**) Test scan obtained by using an intraoral scanner (Co. 3Shape, Trios 4) directly on the model.

10 UCLP models (Co. Smile Train, Cleft Lip Simulator) were scanned using 3 different 3D scanners (Fig. 1A–E). The models simulate the superficial and subcutaneous anatomy of the nasolabial region of a patient with complete left UCLP for training purpose. Extension of the cleft lip in the transversal was a maximum of 5 mm. The scanning of each modality, the master model included, was performed by a different person experienced in the use of the scanners in daily clinical or laboratory practice, including scanner calibration according to the manufacturer's instructions. Each scan within a modality was performed by the same person for better comparability.

R v.4.2.2 with RStudio 2023.03.0 package Optimal design of experiments (OPDOE 1.0-10) was used to determine sample size using triangular testing^9^. To detect two-tailed differences between FS and IOS for integrated and mean distance, α = 0.05, β = 0.2 (corresponding to 80% power)^10^ was chosen. After nine comparisons (n = 18), sequential testing could be stopped in favour of H1. To be on the safe side, 10 comparisons (n = 20) were included in order to be able to detect meaningful differences.

### Master model acquisition

An optical non-contact blue light scanner (Co. GOM, ATOS Triple Scan) was used as previously described by Matta et al. to create a 3D master model of each UCLP model for reference^11,12^ (Fig. 1A,B). First, the models were treated with a sublimating matting spray (Co. AESUB, Blue Scanning Spray). Subsequently, the models were prepared with self-adhesive standardised reference points (Co. GOM) in order to align the generated data in 3D space^13^. Probing error was 4 μm as the measuring volume was beneath 46 mm × 46 mm × 13 mm at all times^14^. The 10 digital master models thus obtained were saved in stereolithography (STL) format. Scanning time was approximately 5 min per model.

### Facial scan acquisition

The facial scanner (Co. Canfield, Vectra H2) requires the pupils, glabella and tip of the nose as specific anatomical landmarks of the face to generate 3D images. Due to restriction to the nasolabial region, these are not present on the UCLP models. To sufficiently apply the facial scanner to the models, they were sequentially embedded in a 3D printed sample face, which was specifically designed and printed for this application (Fig. 1C). In order to realize the sample face, a 3D file of one of the models was subtracted by boolean difference from the 3D scan of a bust of the roman emperor Caracalla (STL file freely available). The 3D file of the model was created using in-domo cone beam computed tomography. Boolean difference and the subsequent modelling were realised with the help of a 3D processing tool (Co. Autodesk, Meshmixer). Each scan was then performed according to a set procedure. The image data generated during this process was stitched into 3D files using the company's own software (Co. Canfield, Vectra) and exported in STL format. Scanning time was approximately 5 min per model.

### Intraoral scan acquisition

In addition, each UCLP model was scanned using an intraoral scanner (Co. 3Shape, Trios 4). A respective 3D file was generated and exported in STL format Fig. 1A,E. The intraoral scanner was used according to standardised procedures (manufacturer's instructions). Scanning time was approximately 2 min per model.

### Analysis

**Figure 2:**
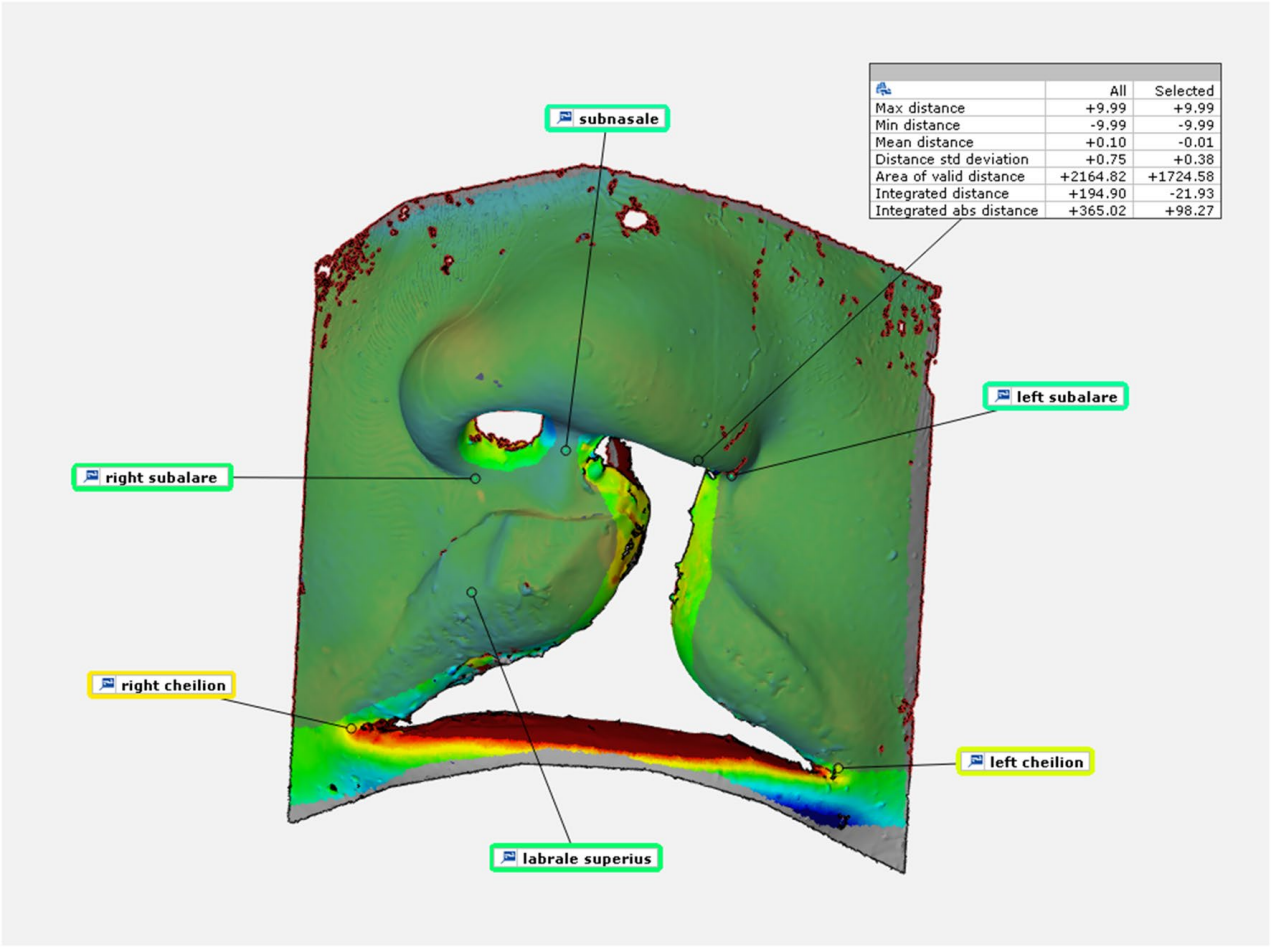
Example of metric surface and point comparison. Surface and point comparison for marked areas; intraoral scanner to master model.

All examinations and analyses were carried out by a single person. The data generated in the form of STL files was compared using a 3D evaluation and measurement tool (Co. GOM, GOM Inspect). Evaluation was performed blinded and in a random order. Both IOS and FS scans were tested against the reference scans. Methodologically, the 3D models were cropped to the size of the area of interest and then aligned using a section-based best-fit algorithm (1.5 mm). The surface comparisons were referenced to the CAD reference with a maximum value of 0.5 mm and a minimum of  − 0.5 mm for all scans. In order to eliminate the effects of distortion of the model when inserted into the sample face for FS testing, the mobile areas of the lower lip and the nostrils were excluded by examining defined, selected areas within the surface comparison. For evaluations, corresponding surface comparisons were made for each model with the master model as the reference plane or CAD reference. The maximum distance, minimum distance, mean distance, mean absolute distance, area of valid distance, integrated distance, and integrated absolute distance parameters were recorded for the marked areas (Fig. 2 and Table 1). Mean distance and integrated distance served as main parameters for evaluation. Mean distance describes the average surface increase in mm, calculated over the average deviation of all points on the mesh. Integrated distance describes the average increase in volume from the arithmetic mean value of the distances of the two surfaces to be compared in mm3 (Integrated distance = ∑ (di*Si); surfaces as “S” and the deviations as “d”). For additional evaluation, deviation flags were placed at prominent points of the nasolabial region including subalare*2, subnasale, labrale superius and cheilion*2 (Fig. 2 and Table 2)^15,16^. The comparative results are presented in the form of boxplots for better illustration.

**Table 1:** Examined parameters for surface and volume comparison and resulting average values and sums of the deviations.

Max distance	Maximum distance allowed for surface comparison on marked area
	Mean deviation: 9.98 FS to reference; 9.97 IOS to reference. ∑: 99.84 FS to reference; 99.65 IOS to reference
Min distance	Minimum distance allowed for surface comparison on marked area
	Mean deviation: − 9.95 FS to reference; − 9.80 IOS to reference. ∑: − 99.47 FS to reference; − 98.02 IOS to reference
Mean distance	Mean distance on marked surface area between “mesh” and “CAD”
	Mean deviation: − 0.07 FS to reference; 0.00 IOS to reference. ∑: − 0.70 FS to reference; − 0.02 IOS to reference
Mean absolute distance	Mean absolute distance on marked surface area between “mesh” and “CAD”
	Mean deviation: 0.09 FS to reference; 0.02 IOS to reference. ∑: 0.90 FS to reference; 0.20 IOS to reference
Area of valid distance	The marked reference surface area that is used for the comparison
	Mean deviation: 1647.41 FS to reference; 1749.66 IOS to reference. ∑: 16474.05 FS to reference; 1749.66 IOS to reference
Integrated distance	The marked inscribed volume of the surface comparison
	Mean deviation: − 146.19 FS to reference; − 8.81 IOS to reference. ∑: − 1461.89 FS to reference; − 88.14 IOS to reference
Integrated absolute distance	Describes an absolute value of the marked integrated distance
	Mean deviation: 450.46 FS to reference; 127.27 IOS to reference. ∑: 4504.56 FS to reference; 1272.71 IOS to reference

Parameters used for area analysis relating to the marked area: Maximum distance; minimum distance; mean distance; mean absolute distance; area of valid distance; integrated distance and integrated absolute distance. All values in the table are given in millimetres (mm).

**Table 2:** Examined parameters for point comparison.

Subalare*2	Point on the margin of the base of the nose where the ala disappears into the upper lip skin
Subnasale	Midpoint of angle at the columella base where the lower border of the nasal septum and the surface of the upper lip meet
Labrale superius	The midpoint of the vermilion line of the upper lip
Cheilion*2	Point located at the corner of each labial commissure

Parameters/Landmarks used for point analysis: Subalare*2; Subnasale; Labrale superius; Cheilion*2.

## Results

**Figure 3:**
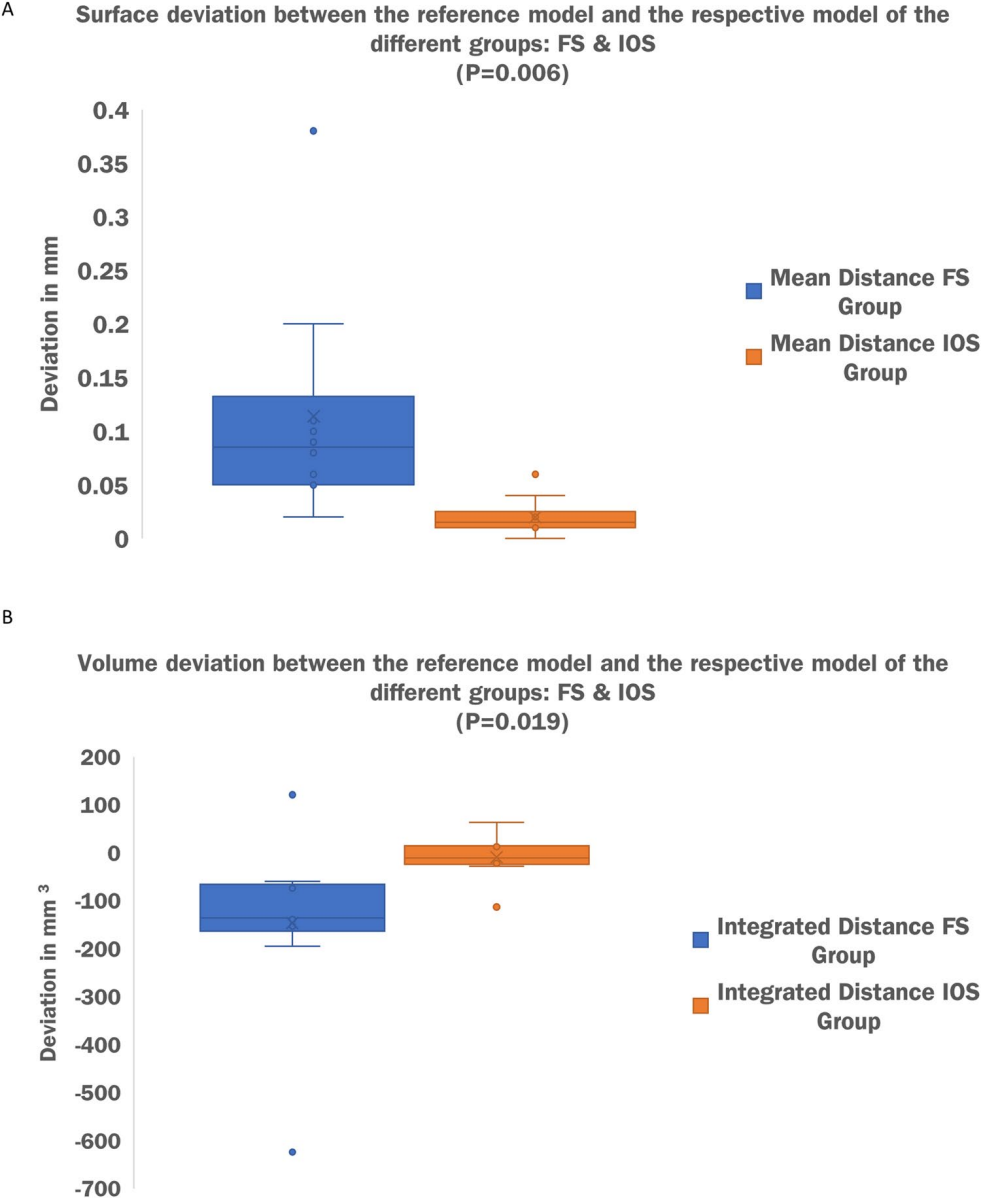
Comparative results for the two main parameters mean distance and integrated absolute distance presented in the form of boxplots. (**A**) X-axis: modality; FS versus Reference followed by IOS versus Reference. Y-axis: mean distance in mm. (**B**) X-axis: modality; FS versus Reference followed by IOS versus Reference. Y-axis: integrated distance in mm3.

**Figure 4:**
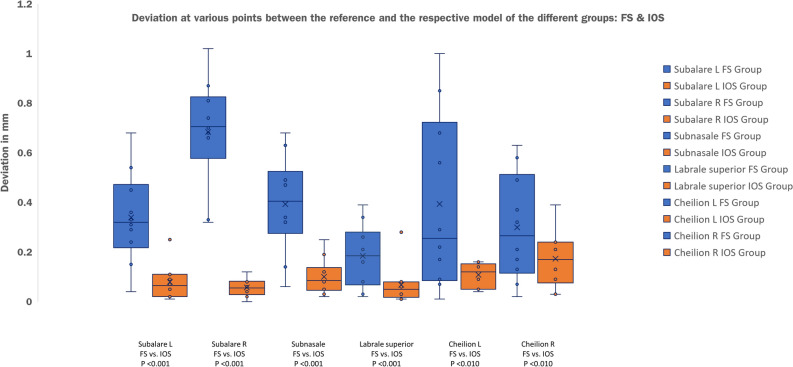
Additional point comparison presented in the form of boxplots. X-axis: modality; FS versus Reference followed by IOS versus Reference as pairs of two. Subsequent listing of point comparisons for: Left subalare, right subalare, subnasale, labrale superior, left cheilion and tight cheilion. Y-axis: integrated distance in mm3.

The FS and IOS metric deviations from a validated reference scanner were tested using surface comparisons with mean distance and integrated distance as the main parameters and point comparisons as additional parameters. Al values in the following are given in millimetres (mm). Comparing the FS to the reference minimum mean distance was 0.02; maximum 0.38 with an average of 0.114. Integrated distance was measured at a minimum of  − 623.89; a maximum of 121.55 with an average of  − 146.189. For surface comparison between the IOS and the reference minimum mean distance was 0.00; maximum was 0.06 with an average of 0.02. Integrated distance was at a minimum of  − 112.83; a maximum of 63.93 and an average of  − 8.814. For better illustration, comparative results for the two main parameters mean distance and integrated distance are presented in the form of boxplots (Fig. 3A,B). Average and summed deviations for the main and secondary parameters are additionally given in Table 1. Point comparison by means of deviation flags resulted in a mean deviation of 0.34, 0.68, 0.39, 0.18, 0.39 and 0.30 for left and right subalare, subnasale, labrale superius and left and right cheilion comparing the FS to the reference. Examination of the IOS compared to the reference resulted in a mean deviation of 0.08, 0.06, 0.10, 0.07, 0.14 and 0.17 regarding the same parameters. The summed deviation of the 6 deviation flags for all 10 model scans is 22.93 for the FS and 6.21 for the IOS compared to the reference. Additionally, results for point comparison are illustrated in Fig. 4. Two sample t-test for unconnected samples showed a significant difference in mean distance (*P* = 0.006) and integrated distance (*P* = 0.019) between both test groups (FS vs. reference and IOS vs. reference). Statistical tests were performed using IBM SPSS (version 28) and by a separate person. Full data are available on request.

## Discussion

Previously, an industrial, non-contact white light scanner (Co. GOM, ATOS III Triple Scan) was validated as a highly precise measurement technique down to the micrometric scale^11^. However, due to its stationary character the scanner is not applicable in the clinical setting.

As precision of dental impressions is crucial for the success of dental restoration^17^, dental medicine places high demands on the metric accuracy as well as the mobility of IOS. Recent data confirms high accuracy for current generation mobile IOS in dental application^18^. Data on current generation handheld face scanners for facial 3D assessment is inconsistent^19,20^.

In search of a metrically accurate and practical instrument for 3D survey of the nasolabial region, we encountered facial scanners and, in the following, the Vectra H2 scanner (Co. Canfield), which is promoted for the assessment of plastic facial surgery. Simultaneous literature research identified the possibility for extraoral use of modern intraoral scanners^21^.

To enable solid clinical data collection and research, both options now available required testing against a validated baseline. For intraoral use, IOS have been tested against a validated reference^22,23^. For extraoral use, Ayoub et al. validated the mobile IOS (Co. 3Shape, Trios 3, Copenhagen, Denmark) as a reliable source for measuring lip asymmetry and scar surface and described the scanner as a useful tool for routine clinical use and for objective outcome measurements in surgical repair of cleft lip^14^. Results were presented on the basis of moderate correlation between scan data and subjective visual assessment. Handheld facial scanners (FS) are heavily promoted for evaluation of plastic facial and orthognathic surgery. Regarding recent evaluation of facial scanners for metric accuracy, stereophotogrammetric 3D reliability tests were conducted without comparison to a previously validated baseline, so possible inaccuracy cannot be excluded^24^.

To the best of our knowledge and based on current literature, for the first time we present further validation for the tested IOS and FS on 3D assessment of the nasolabial region by adding the previously validated ATOS III Triple Scan blue light scanner as a reference^11^. Since direct testing on patients is not practical due to the stationary nature of the reference scanner and the natural facial movements of awake patients, we decided to perform the validation test on models to allow comparison with a validated baseline. Applying the FS scanner on UCLP models required creative ways of overcoming technical hurdles. Since in vitro cleft palate models cannot be captured with the facial scanner due to a lack of anatomical landmarks such as the pupils, we used advanced 3D modelling and printing by embedding the cleft models one by one into a 3D-printed sample face based on a bust of the ancient Roman Emperor Caracalla (Fig. 1C). To minimize improvements in scan quality due to individual learning as a confounding factor, each modality (IOS, FS, reference) was scanned by a different individual. Scans within each modality were performed by the same person.

Different study’s used a specific number of anatomical landmarks on the face without disclosing the regional asymmetry between these landmarks for UCLP assessment^25,26^. As this allows for errors in the 3D analysis of the face^15^, additional surface comparisons were conducted as a basis for evaluation.

Virtual superimposition can be performed using various software. Surface comparisons are performed on the basis of best-fit algorithms, whereby the choice of algorithm influences the alignment discrepancy^27–30^. Entire dataset or section-based best-fit algorithms achieve the best results according to recent studies^31^. Considering this current literature which matched our own experience, we decided to perform the surface comparisons on the basis of a section-based best-fit algorithm. Hereafter, mean deviation value is a useful and frequently used tool for quantifying the difference between two meshes, providing valuable information for improving the quality and accuracy of 3D model scans^32^. Since current literature incorporates additional calculation of the integrated distance, which can be described as the difference in volume between the two meshes, the parameter was included^32,33^.

The main limitation of the study is the translation of ex vivo data to an in vivo application, which in this case was necessary for the methodological reasons mentioned above. To keep the test as close as possible to the scanned morphology in a clinical setting, high-fidelity cleft surgery simulators were used as scanned subjects. In addition, the distortion of the soft silicone model when placed in the model face must be considered as a limiting factor. As mentioned in the analysis section, this was addressed by excluding the mobile areas of the lower lip and nostrils from the surface comparisons in order to avoid consequential errors and effects on the measurement series.

In addition to the data-based evidence, discrepancies in scan quality are also evident by visual inspection (Fig. 1D,E). Observation of the data and direct scans allows us to make assumptions about the qualitative difference between the intraoral and facial scanners examined. Both scanners under review differ fundamentally in terms of their methodology and functioning. Intraoral scanners project a light beam onto the surface to be analysed; consecutively the lights deformation on such surfaces is captured by two or more cameras (often times processing thousands of frames per second) and exploited for the calculation of 3D coordinates^34–36^. In contrast to the continuous image acquisition and processing of the intraoral scanner, the facial scanner under investigation operates by “stitching” together 3 individual images. Strong interpolation during image stitching might be the cause of inaccuracies in surface imaging and the resulting inadequacy for clinical-scientific use.

We successfully demonstrated that the investigated FS and IOS differ significantly in terms of their metric precision when scanning cleft models (significantly higher precision for the IOS) when compared to a validated baseline. Validation studies for dental use of the studied IOS, using similar methodology, yielded comparable, even slightly inferior results in terms of average accuracy (0.06 mm in the dental setting vs. 0.02 in the extraoral/cleft setting)^37^. Regarding the clinical significance of the measurement deviations compared to the reference, it can be assumed that the intraoral scanner, due to its significantly lower metric deviation in the experimental setting, also provides an accurate image of the nasolabial region of cleft patients in clinical use. In terms of predictable clinical applicability of each scanner, the IOS not only outperforms the FS in metric accuracy, but also takes less than half the time to scan. Therefore, its implementation would be an important step in the short- and long-term 3D documentation of cleft patients and would enable new ways of surgical evaluation. As the next step, clinical applicability of the IOS in the assessment of the nasolabial region must now be established in both healthy patients and patients with cleft-related facial deformities.

## Conclusions

Compared to the FS and with reference to a validated baseline scan, the IOS shows significantly higher metric precision in cleft model scanning. Although limited by the translation from the *ex* to the in vivo setting, the data collected provides a sound basis for the clinical application of the IOS in 3D assessment of the nasolabial region. Further clinical trials are recommended for the use of IOS in the assessment of cleft lip and palate surgery.

## Data Availability

Additional data on primary and secondary parameters recorded are available on request. For enquiries, please contact the corresponding author at rainer.lutz@uk-erlangen.de.

## Abbreviations

3D: 3-Dimensional
FS: Facial scanner
IOS: Intraoral scanner
UCLP: Unilateral cleft lip and palate
STL: Stereolithography
